# Enhanced screening for tuberculosis infection among immigrants in southern New Brunswick: A cross-sectional pilot study

**DOI:** 10.14745/ccdr.v51i05a03

**Published:** 2025-05-01

**Authors:** Isdore Chola Shamputa, Duyen Thi Kim Nguyen, Hope Mackenzie, Derek J Gaudet, Alicia Harquail, Kim Barker, Duncan Webster

**Affiliations:** 1 Department of Nursing and Health Sciences, University of New Brunswick, Saint John, NB; 2 Department of Health, Government of New Brunswick, Saint John, NB; 3 Faculty of Business, University of New Brunswick, Saint John, NB; 4 Microbiology Laboratory, Saint John Regional Hospital, Saint John, NB; 5 Department of Psychology, University of New Brunswick, Saint John, NB; 6 Dalhousie Medicine New Brunswick, Dalhousie University, Saint John, NB; 7 Division of Medical Microbiology, Department of Laboratory Medicine, Saint John Regional Hospital, Saint John, NB; 8 Division of Infectious Diseases, Department of Medicine, Saint John Regional Hospital, Saint John, NB

**Keywords:** tuberculosis infection, screening, prevention, immigrant health, IGRA

## Abstract

**Background:**

In 2021, approximately 77% of active tuberculosis (TB) disease (TBD) cases in Canada were among foreign-born individuals. Less than 3% of TBD cases in Canada are detected through pre-arrival Canadian immigration medical examinations (i.e., chest X-rays), and the remaining 97% are likely due to reactivation of undiagnosed latent TB infection (TBI) post-arrival. In New Brunswick, the proportion of TBD cases among foreign-born individuals gradually increased from about 33% (1/3 individuals) in 2013 to 100% (14/14 individuals) in 2023. The objective of this study was to estimate the prevalence of TBI among immigrants in southern New Brunswick, identify potential predictors for positive TBI screening and assess participant experiences with the pilot TBI screening procedure.

**Methods:**

A cross-sectional study was conducted from November 2021 to November 2023 among immigrants ≥19 years old who had no history of TBD and were born in a country with a TB incidence rate of ≥40/100,000 population or were referred by healthcare professionals. Participants were recruited through various channels and underwent TBI screening using the interferon-gamma release assay, followed by a survey on their screening experience.

**Results:**

Of the 264 participants, 49 (18.6%) screened positive for TBI. Factors associated with higher odds of screening TBI-positive included birthplace in a “highly to severely endemic” (≥300/100,000 population) TB-incidence country (OR=3.24; 95% CI: 1.07–9.81) and increased age (OR=1.05; 95% CI: 1.01–1.08). Participants rated the pilot TBI screening procedure positively (mean scores ranged from 4.03–4.55 on a five-point Likert scale).

**Conclusion:**

Results suggest that immigrants born in countries with TB incidences of ≥300/100,000 population should be considered for screening and treatment of TBI. The pilot TBI screening procedure yielded positive feedback. Further research with a larger sample is recommended.

## Introduction

Approximately 25% of the global population has latent tuberculosis (TB) infection (TBI) ((1,2)), of which 5%–10% go on to develop active TB disease (TBD) (3–5). In 2023, there were 10.8 million TBD cases and 1.25 million TBD-related deaths worldwide (5). While more than 80% of the TBD cases and deaths occur in low- and middle-income countries ((5)), high-income countries also report TBD cases, in part due to immigration and international travel ((6–9)).

In 2021, Canada had a TBD incidence rate of 4.8 cases per 100,000 population and 76.7% of the cases were among individuals born outside Canada ((10)). The Atlantic Canadian province of New Brunswick (NB) has experienced a notable rise in immigration in the last decade ((11,12)). Coinciding with this immigration growth is the increase in the proportion of TBD cases among foreign-born individuals. For example, the proportion of foreign-born TBD cases has gradually risen from 33% (n=1) in 2013 to 100% of cases in 2022 (n=17) and 2023 (n=14) (*personal communication, Public Health Agency of Canada TB Task Force Meeting, November 26–27, 2024*). The rise of TBD in NB provides an opportunity-for the provincial healthcare system to explore additional preventative strategies to curb the increase of TBD and protect and promote the health of its population, while continuing to welcome new immigrants to the province.

Current pre-arrival Canadian immigration medical examinations focus on detecting TBD through screening with chest X-ray ((13)). The current screening system does not assess for TBI, thus creating a gap where individuals with TBI are at-risk of TB reactivation post-arrival. Research has shown that <3% of TBD cases diagnosed among immigrants to Canada are detected through the immigration post-landing surveillance program ((14)). In 2019, Immigration, Refugees and Citizenship Canada (IRCC) broadened screening requirements to include pre-arrival TBI screening for certain high-risk immigration applicants (**Box 1**) ((13)). To our knowledge, this program has not been evaluated and the number of TBD cases averted is unknown. While the TB incidence rate of a person’s country of birth has been shown to be a risk factor for TBI ((15–18)), it is noticeably absent from the recent IRCC recommendations ((13)). Interestingly, there are currently no widely established avenues for routine TBI screening for immigrants in Canada, despite its potential to curb increasing prevalence of TBD, and the call from experts for enhanced screening efforts ((19)).

Box 1 **Immigration, Refugees and Citizenship Canada screening criteria for tuberculosis infection^a^**

**Screening criteria for tuberculosis infection**

1. Individuals seropositive for human immunodeficiency syndrome

2. Individuals who have been in close contact with TB disease in the last five years

3. Individuals with a history of certain head and neck cancers within the previous five years

4. Individuals undergoing dialysis or suffering from advanced chronic kidney disease

5. Individuals who have had solid organ or bone marrow transplants and are receiving immunosuppressive therapy

^a^ Adapted from Immigration, Refugees and Citizenship Canada. Canadian panel member guide to immigration medical examinations Immigration, Refugees and Citizenship Canada. Ottawa, ON: Government of Canada. https://www.canada.ca/en/immigration-refugees-citizenship/corporate/publications-manuals/panel-members-guide.html

The purpose of this study was to enhance current TBI screening in southern NB, which presently follows the IRCC TB screening protocol, by investigating the potential value of a targeted TBI pilot screening program among immigrants in southern NB. This study seeks to address the gap from IRCC’s current TB screening system, by estimating the TBI prevalence among immigrants in southern NB, identifying potential predictors of screening positive for TBI and evaluating the experience of immigrants taking part in the pilot TBI screening procedure. To our knowledge, this is the first Canadian study to offer routine TBI screening to all immigrant streams (e.g., temporary foreign workers, family reunion, permanent residents, international students, refugees), whereas in previous Canadian TBI studies, the primary focus was on the refugee populations ((20–23)).

The interferon-gamma release assay (IGRA) was used for TBI screening in this study instead of the traditional tuberculin skin test (TST). The IGRA offers several advantages, including the use of antigens more specific to *Mycobacterium tuberculosis*, a single visit for blood draw, screening not influenced by the Bacille Calmette−Guérin (BCG) vaccination or previous exposure to certain non-tuberculous mycobacteria and improved consistency in results with fewer concerns regarding inter-rater reliability ((24,25)).

## Methods

### Study design

A cross-sectional study was conducted among immigrants to Canada, from November 4, 2021, to November 21, 2023. Participants were eligible if they were ≥19 years old and resided in southern NB, and were either: a) born in a high-TB-incidence country, defined as ≥40 cases per 100,000 population) ((7)), b) were referred to Public Health NB by IRCC or c) were at high risk of TBI due to having a TBD-positive partner or having lived in a high-TB-incidence country, or were considered to be at high-risk of TBI following the post-arrival health assessments (PAHAs) of government-assisted refugees (GARs) by NB Primary Health Care. Participants with a history of TBD or TBD treatment were excluded from this study.

### Participant recruitment

Participants were recruited using posters, social media channels, snowball sampling, public health channels or PAHAs of GARs. Prospective participants contacted the research team using the phone number or email provided on recruitment materials or met with a research team member in-person after their PAHAs. To help minimize potential recruitment bias, this study included all eligible immigrants at risk of TBI, irrespective of the language they spoke, when they arrived in Canada, or their immigration stream.

Sample size was estimated *a priori* as previously described (26,27). Based on an estimated prevalence of TBI of 25% ((2)), our calculation yielded an estimated minimum sample size of 160 individuals. The published study protocol describes a minimum sample size estimate of 240 individuals, but this discrepancy is due to removing two predictors (i.e., comorbidities and immigration stream) ((28)). Co-morbidities were removed because they were not feasible to collect due to lack of access to personal medical records, and immigration stream was omitted because it resulted in too many dummy variables, which would have reduced the power of the analysis. Due to the aforementioned reasons, only four of the six original variables were included in the study’s regression analyses (i.e., age, sex, gender and incidence rate classification).

### Procedure

Potential participants were asked to provide demographic information, including sex, gender, date of birth, country of birth, date of arrival in Canada and type of Canadian entry visa to assist with screening for eligibility.

### Pilot tuberculosis infection screening process

Those that met the study’s eligibility criteria were provided a consent form to review. Consent forms were translated into the seven most spoken languages among immigrants in southern NB ((29)). Following written consent, a research member organized phlebotomy appointments at one of two local hospitals, chosen by the participant. During phlebotomy, hospital staff guided participants through established protocols. For participants recruited at PAHAs, the study was explained by a research team member in their chosen language with the help of a virtual translation service. The YMCA of Greater Saint John staff, which is the local immigrant-serving organization responsible for GAR settlement, arranged phlebotomy appointments and accompanied participants. Other community partners, such as the Saint John Newcomers Centre and PRUDE Inc., also offered close collaboration to support study participants.

### Materials

All blood samples were transported to the Saint John Regional Hospital Microbiology Laboratory for TBI screening. Screening was performed using the IGRA (QuantiFERON-TB Gold Plus; QIAGEN, Germantown, Maryland, United States [US]) as per the manufacturer’s recommendations. The IGRA results were categorized as either positive (≥0.35 IU/mL), negative (<0.35 IU/mL) or indeterminate ((29)). An IGRA was repeated if samples yielded an indeterminate result. If the result remained indeterminate, next steps were determined through review and clinical assessment by an infectious disease specialist. The IGRA results and relevant diagnostic data were accessed and communicated to participants by a healthcare provider. Participants positive for TBI were offered clinical assessment. In cases, where the IGRA was positive, TBD was ruled out and participants were offered TB preventive treatment (TPT). The TPT results will be provided in a follow-up paper.

Following the screening procedure, participants were asked to complete a TBI pilot screening process experience survey using Qualtrics, an online survey platform. The survey was intended to gather information regarding their experience of participating in this study, and included 11 questions on five-point Likert scales, along with open-ended questions. Participants completed the survey virtually through an email containing an attachment or a link to the survey, provided by a research team member. The survey questions focused on 1) equity, diversity, and inclusion; 2) barriers and facilitators to TBI screening, such as language, cultural factors, accessibility of the blood collection facility, interactions with healthcare professionals; and 3) the overall ease or difficulty of participating in the study (see **Appendix 1**) (28).

### Data analysis and management

The TB incidence rate of a participant’s country of birth was obtained through World Health Organization (WHO) data ((30)) to create incidence rate categories. Participants were classified as being born in countries with incidence rates considered to be low (<10/100,000 population), lower-moderate (10–49/100,000 population), upper moderate (50–99/100,000 population), endemic (100–299/100,000 population), highly endemic (300–499/100,000 population) or severely endemic (≥500/100,000 population), as per the WHO classification (31). Due to low counts in certain categories, and to maintain adequate statistical power, incidence categories were combined into three new categories for the analyses: “sub-endemic” (0–99/100,000 population), comprising low, lower-moderate and upper moderate, and “highly to severely endemic” (≥300/100,000 population), comprising highly endemic and severely endemic. The “endemic” category (100–299/100,000 population) remained unchanged (31).

In terms of data management and analysis, survey data was completed in Qualtrics, exported to Excel and merged with participants’ demographic data; thereafter, the data was de-identified and stored on an encrypted OneDrive account. The quantitative de-identified data was imported into IBM SPSS Statistics (version 29; Armonk, New York, US) for analysis. Categorical measures were presented as frequencies and percentages, and continuous measures were presented using means and standard deviations (SD). Associations between predictors and TBI were presented as odds ratios (ORs) with 95% confidence intervals (CIs).

For the sensitivity analysis, literature suggests that recent immigrants are at a higher risk of developing TBD from TBI within the first two to five years of their arrival ((32)); thus, binary logistic regression analysis was repeated using data from those that had arrived in the last five years, as this cohort would benefit most from being screened and treated.

### Ethical approval

This study was approved by the Horizon Health Network (file #: RS 2021–3046) and University of New Brunswick (file #: 033-2021) Research Ethics Boards.

## Results

### Participants

**Figure 1:**
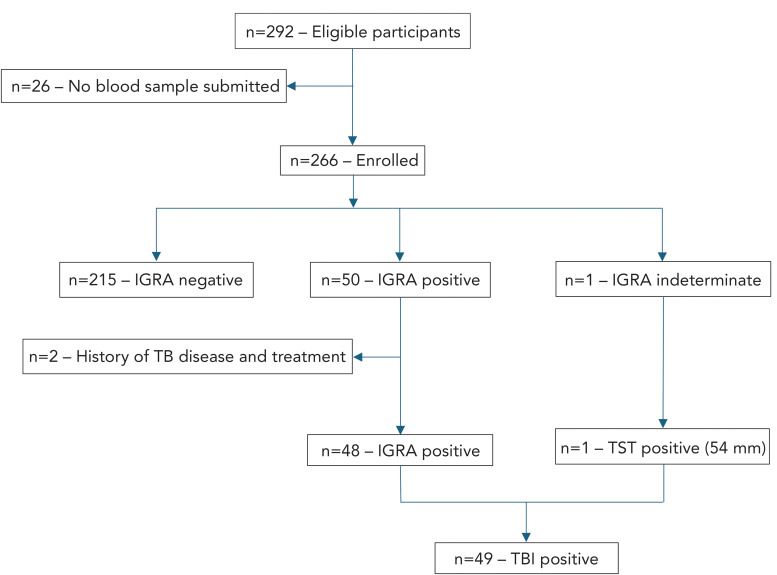
Participant flowchart Abbreviations: IGRA, interferon-gamma release assay; TB, tuberculosis; TBI, tuberculosis infection; TST, tuberculin skin test

Of 292 participants who consented to this study, a total of 28 participants were excluded as they had not submitted blood specimens for TBI screening (n=26) or had a previously undisclosed history of TBD and treatment (n=2). Among 26 participants who did not provide blood samples for TBI screening, 17 were born in “endemic” to “highly to severely endemic” TB incidence countries, of whom 16 (94.1%) relocated to other provinces. After excluding the aforementioned participants, the total sample size was 264 (**Figure 1**).

The average age of the 264 participants was 36.8 years (SD=10.27), ranging from 19 to 67 years. More than half (53.8%) identified as female. Self-reported biological sex and gender identity was consistent across participants, except for one participant who identified their biological sex as male and identified their gender as a trans-female. Given the near-identical count for biological sex and gender identity, sex was retained in the regression analyses, while gender was dropped. Additional descriptive characteristics are presented in **Table 1**.

**Table 1:** Descriptive statistics of participants (n=264)

Characteristic	Variables	TBI		n
		Positive^a^ (%)	Negative (%)	
Sex	Male	21 (17.2%)	101 (82.8%)	122
	Female	28 (19.7%)	114 (80.3%)	142
	Total	49 (18.6%)	215 (81.4%)	264
Age group (years)	19‒24	3 (8.6%)	32 (91.4%)	35
	25‒34	11 (14.1%)	67 (85.9%)	78
	35‒44	22 (22.0%)	78 (78.0%)	100
	45‒54	8 (21.1%)	30 (78.9%)	38
	55‒64	4 (36.4%)	7 (63.6%)	11
	65 and older	1 (50.0%)	1 (50.0%)	2
	Total	19 (7.2%)	215 (81.4%)	264
Year of arrival in Canada	2000	0 (0.0%)	1 (100%)	1
	2001	1 (100%)	0 (0.0%)	1
	2002	0 (0.0%)	1 (100%)	1
	2007	0 (0.0%)	3 (100%)	3
	2010	0 (0.0%)	4 (100%)	4
	2012	0 (0.0%)	2 (100%)	2
	2013	0 (0.0%)	2 (100%)	2
	2014	1 (100%)	0 (0.0%)	1
	2015	0 (0.0%)	1 (100%)	1
	2016	1 (33.3%)	2 (66.7%)	3
	2017	0 (0.0%)	7 (100%)	7
	2018	1 (10.0%)	9 (90.0%)	10
	2019	3 (16.7%)	15 (83.3%)	18
	2020	0 (0.0%)	6 (100%)	6
	2021	5 (16.1%)	26 (83.8%)	31
	2022	18 (19.4%)	75 (80.6%)	93
	2023	19 (23.8%)	61 (76.2%)	80
	Total	49 (18.6%)	215 (81.4%)	264
Country of birth by World Health region	Western Pacific	7 (20.6%)	27 (79.4%)	34
	Americas	4 (16.7%)	20 (83.3%)	24
	Africa	11 (24.4%)	34 (75.6%)	45
	South-East Asia	2 (6.7%)	28 (93.3%)	30
	Europe	0 (0.0%)	8 (100%)	8
	Eastern Mediterranean	25 (20.3%)	98 (79.7%)	123
	Total	49 (18.6%)	215 (81.4%)	264
TBI screening results by recruitment category	Born in high TB incidence country^b^	44 (20.4%)	172 (79.6%)	216
	Referred by Public Health^b^	1 (50.0%)	1 (50.0%)	2
	Recruited via PAHAs^b^	4 (10.3%)	35 (89.7%)	39
	At-risk of TBI^c^	0 (0.0%)	7 (100%)	7
	Total	49 (18.6%)	215 (81.4%)	264
TB incidence by country of birth (per 100,000 population)	Sub-endemic (0–99)	15 (13.8%)	94 (86.2%)	109
	Endemic (100–299)	25 (20.0%)	100 (80.0%)	125
	Highly to severely endemic (≥300)	9 (30.0%)	21 (70.0%)	30
	Total	49 (18.6%)	215 (81.4%)	264
Primary healthcare provider (doctor/nurse practitioner) in Canada	No	49 (21.7%)	177 (78.3%)	226
	Yes	0 (0.0%)	38 (100%)	38
	Total	49 (18.6%)	215 (81.4%)	264

Abbreviations: PAHA, post-arrival health assessment; TB, tuberculosis; TBI, tuberculosis infection

^a^ Includes one individual with an indeterminate IGRA and positive tuberculin skin test

^b^ ≥40/100,000 population

^c^ Due to TBD-positive partner or having lived in a high-TB-incidence country (born in a low-TB-incidence country, i.e. <40/100,000 population)

### Tuberculosis infection screen outcome

Of 264 participants, 18.6% (n=49) had a positive TBI screening. One participant had an indeterminate IGRA result on two separate screens due to a reactive negative control. Subsequent TST in the indeterminate case revealed an induration of 54 mm. Based on the participant’s demographics, clinical evaluation and radiological studies, this participant was categorized as TBI-positive (Figure 1). Table 1 includes TBI screening results by sex, age range, year of arrival in Canada, country of birth by WHO region, recruitment category, TB incidence of country of birth and primary healthcare provider.

When comparing IGRA screening results by recruitment categories, most of the positive results were found among immigrants born in high-TB-incidence countries. Similarly, when analyzed based on TB incidence rate classifications, a higher proportion of participants born in “highly to severely endemic” TB countries screened positive for TBI (30%), compared to those born in TB-“endemic” (20%) and “sub-endemic” (14%) countries (Table 1).

### Binary logistic regression

Binary logistic regression was used to predict TBI screen outcome using age, sex and TB incidence category by country of birth as predictors. Odds ratios were examined to determine the impact of each variable. The binary logistic regression model was statistically significant, χ^2^ (4)=13.42, *p*=0.009, with a Nagelkerke R^2^ value of 0.08. The odds of a positive screen were approximately 3.5 times higher for individuals born in a “highly to severely endemic” country compared to those born in countries classified as “sub-endemic” (OR=3.45; 95% CI: 1.28–9.27). Also, age was found to increase the odds of a positive TBI screen (OR=1.05; 95% CI: 1.02–1.08). Neither sex, nor birth in a TB “endemic” country, was found to be statistically significant (**Table 2**). Sensitivity analyses results were consistent with the main results (**Appendix 2**, **Table A1**).

**Table 2:** Logistic regression analysis of positive tuberculosis infection screen results

Predictor variable	ß	SE	*p*-value	Odds	95% CI for odds ratio	
					Lower	Upper
Age	0.048	0.016	**0.003**	1.049	1.016	1.083
Sex	−0.106	0.329	0.747	0.899	0.472	1.713
Endemic	0.516	0.364	0.156	1.675	0.821	3.421
Highly to severely endemic	1.237	0.505	**0.014**	3.446	1.280	9.272

Abbreviations: CI, confidence interval; SE, standard error

Note: Model χ^2^=13.42, *p*=0.009, Nagelkerke R^2^=0.08, N=264. The dependent variable in the analysis was coded as 0=negative result and 1=positive result. Bolded *p*-values are statistically significant

### Participant experience of the tuberculosis infection screen process

To assess the participants’ experience in the TBI pilot screening procedure, means and SDs were calculated to assess each survey item response and interpreted with guidance described previously ((33)). Surveys regarding the pilot TBI screening procedure were completed by 176 participants (66.7 overall response rate), with more than half (54.1%) being self-reported female respondents. The mean age was 36.78 years (SD=9.75). Participant responses are presented in **Table 3**. Participants rated the ease of locating the phlebotomy site most favourably (M=4.55, SD=0.68) and wait times for phlebotomy least favourably (M=4.03, SD=1.03). Participants reported positive attitudes towards TB and expressed willingness to recommend TBI screening to others.

**Table 3:** Survey ratings regarding the pilot tuberculosis infection testing procedure

Item	N	Mean	SD
I received information on why the latent tuberculosis test was being done.	153	4.52	0.61
The blood collection office was easy to find.	172	4.55	0.68
The blood collection office was easy to travel to.	171	4.53	0.61
The blood collection process was simple (e.g., registration, blood collection).	172	4.49	0.64
The waiting time for blood collection was reasonable.	166	4.03	1.03
The healthcare provider (i.e., doctor, nurse) answered all my questions.	168	4.35	0.81
I was satisfied with the overall experience with the latent tuberculosis screening process and/or care I received.	172	4.46	0.77
I would recommend other people to do a latent tuberculosis screening test.	169	4.52	0.65
My knowledge regarding tuberculosis improved by participating in the study.	169	4.12	0.96
My attitudes regarding tuberculosis improved by participating in the study.	167	4.25	0.79

Abbreviation: SD, standard deviation

## Discussion

This pilot study was the first to offer routine TBI screening among immigrants in Atlantic Canada. It differs from previous studies in Canada in several key ways: a) TBI screening was offered to all immigrant groups, rather than focusing on refugee populations; b) it employed the IGRA, in contrast to the traditional TST; and c) the research team collaborated closely with immigrant-serving community partners, which was deemed important for raising awareness and the successful completion of TBI screening and treatment programs in this setting. With respect to the current TB screening practices in NB, our study identified nearly 50 TBI-positive individuals of which only one was referred by IRCC to Public Health NB. These results highlight potential opportunities for increased screening for TBI amongst immigrants in NB.

This study had three key findings. First, a TBI prevalence of 18.6% was found among immigrants in southern NB. Second, increased age and birth in a country with a “highly to severely endemic” TB incidence were identified as factors associated with greater odds of screening positive for TBI. Third, the IGRA worked well in this setting and study participants reported that the pilot TBI screening procedure used in this study was satisfactory.

The prevalence of TBI in this study is comparable to earlier reports of immigrants in other low TB incidence countries ((34,35)), but lower than global and Canadian estimates of 25% ((36,37)). We suspect this difference may be attributed to the local pattern of immigration during the study period and/or the smaller study sample size.

Results regarding the association between birth in a “highly to severely endemic” TB incidence country and TBI are akin to previous research ((35,37,38)). These findings indicate that TBI screening for immigrants born in “endemic” and “sub-endemic” countries may be of less value than screening immigrants from countries with “highly to severely endemic” TB incidence. Likewise, our results associating older age with positive TBI screening are congruent with earlier reports ((39–42)). One plausible explanation for these associations is that older age and high TB incidence in the immigrant’s country of birth increases the participants’ vulnerability for TBI due to greater time to the potential exposure to TBD, emphasizing the need to institute mitigating factors, such as TB awareness campaigns, early detection and TPT to protect and enhance overall health.

It is noteworthy that most participants who relocated to other provinces before providing blood samples for TBI screening were born in “endemic” and “highly to severely endemic” countries. This is concerning, as TBI is not reportable in most public health jurisdictions and cases may go unidentified with the potential for development of TBD. Study participants expressed general satisfaction with the TBI screening process, and the use of the IGRA as the screening tool was a novel component of this study. However, participants desired shorter wait times for sample collection. Additionally, there was a recognized need for increased awareness campaigns aimed at improving understanding and attitudes toward TB.

Regarding the year of arrival and TBI screening, it is important to recognize that immigrants to Canada who have lived in the country for an extended time may travel back to their home country. Although they may have tested negative for TBD during their initial immigration screening, these subsequent visits could be associated with new exposures and the potential for subsequent development of TBD ((43)). However, this would have had minimal impact on our sample, as over three-quarters of study participants arrived within the previous three years.

Use of the IGRA allowed for several distinct advantages in this pilot screening study. Many study participants had a prior history of BCG, which lowers the specificity of the TST ((25,44)). In addition, many participants were recent immigrants with multiple competing obligations and new to a local health system with many barriers. As such, minimizing the complexity and the number of clinic visits was desirable. Many immigrants were undergoing phlebotomy for other PAHA-associated testing and thus the IGRA could be easily incorporated into this process. No additional follow-up reading was required, as would have been the case with the use of the TST ((25)). In a setting of low TB incidence, where proficiency with TST administration and reading may be inadequate, the IGRA provided objective results, avoiding issues of inter-observer variability ((44)). Furthermore, in a post-pandemic setting with strained healthcare resources, a shortage of clinical staff, and a lack of quality assessment with TST, the IGRA can promote efficiency in the use of local resources. The cost effectiveness of the IGRA over the TST in this setting continues to be assessed and will benefit from further study.

In March 2024, the Public Health Agency of Canada established a one-year time-limited TB task group, whose membership includes representatives from each province, territory and national Indigenous organizations. The timeliness of these findings is relevant to the anticipated recommendations to be shared with the Communicable and Infectious Disease Subcommittee of the Public Health Agency of Canada in March of 2025.

### Limitations

Results from this study should be interpreted with caution. First, the study participation acceptance rate was not calculated, due to various recruitment methods, which made it difficult to track those who declined to participate. Thus, feedback regarding participants’ experiences of the pilot TBI screening procedure may be biased towards those who are concerned about TB. Second, this study had a relatively small sample size, and the fluctuating immigration patterns during the study period may limit the generalization and application of the study’s results to inform policy. Also, the study produced fewer positive screening results than the expected number that was based on the global TBI incidence estimate. Although statistical power was maintained, this came at the cost of excluding predictors such as immigration stream. Further, while we intended to include all six WHO TB recommended incidence categories into the model, sample size necessitated the collapse of some categories, resulting in the use of only three broader categories. Future research should aim to include all six recommended categories to establish a more granular understanding of the risk associated with WHO-designated incidence rate categories. Third, data on comorbidities were not gathered, due to challenges in obtaining accurate information. Such data could have offered insights into the risk of early progression from TBI to TBD. More stringent inclusion criteria, refining recruitment methods, and expanding the sample size could help address these limitations. Fourth, the nature of the study limited the ability to account for potential confounding factors, as it was not an experimental study with random sampling or stringent controls. While all immigrants were eligible to participate, only those who volunteered during the recruitment period were included, which may introduce self-selection bias. We recognize the potential for other confounding variables, but addressing them was beyond the scope of this study. Although the regression analysis accounts for age and sex, no additional data were collected to control for other potential confounders.

## Conclusion

This study provides initial insights into the prevalence and contributing factors associated with screening positive for TBI among immigrants in southern NB. Study results highlight the role for screening immigrants from countries with a TB incidence of ≥300/100,000 population using the IGRA. With further refinement, the TBI screening procedure used in this study could be valuable for broader program application.

## Appendix 1 Participant Survey

Instructions: Please circle the response that best describes your agreement with the statement.

1. I received information on why the latent tuberculosis test was being done.

**Table:**

Strongly disagree	Disagree	Neutral	Agree	Strongly agree
1	2	3	4	5

2. The blood collection office was easy to find.

**Table:**

Strongly disagree	Disagree	Neutral	Agree	Strongly agree
1	2	3	4	5

3. The blood collection office was easy to travel to.

**Table:**

Strongly disagree	Disagree	Neutral	Agree	Strongly agree
1	2	3	4	5

4. The blood collection process was simple (e.g., registration, blood collection).

**Table:**

Strongly disagree	Disagree	Neutral	Agree	Strongly agree
1	2	3	4	5

5. The waiting time for blood collection was reasonable.

**Table:**

Too long	Somewhat long	Neutral	Somewhat short	Very short
1	2	3	4	5

6. The healthcare provider (i.e., doctor, nurse) answered all my questions well.

**Table:**

Strongly disagree	Disagree	Neutral	Agree	Strongly agree
1	2	3	4	5

7. I was satisfied with the overall experience with the latent tuberculosis screening process and/or care I received.

**Table:**

Strongly disagree	Disagree	Neutral	Agree	Strongly agree
1	2	3	4	5
**If you were not satisfied, why?**				

8. I would recommend other people to do a latent tuberculosis screening test.

**Table:**

Strongly disagree	Disagree	Neutral	Agree	Strongly agree
1	2	3	4	5
**If you not, please explain:**				

9. My knowledge regarding tuberculosis improved by participating in the study.

**Table:**

Strongly disagree	Disagree	Neutral	Agree	Strongly agree
1	2	3	4	5
**Please explain:**				

10. My attitudes regarding tuberculosis improved by participating in the study.

**Table:**

Strongly disagree	Disagree	Neutral	Agree	Strongly agree
1	2	3	4	5
**Please explain:**				

11. Do you have any suggestions on how the latent tuberculosis testing process can be improved?

**Table:**

Yes	No
1	2
**If yes, please provide your suggestions below:**		

## Appendix 2 Sensitivity analysis

Analyses were conducted using one additional strategy. As research has shown that recent immigrants are more at risk of developing tuberculosis (TB) disease (TBD) from remotely acquired TB infection (TBI), we decided to run the binary logistic regression using data from immigrants who had arrived in the last five years (i.e., since 2019). There was no significant change in the pattern of results. Two hundred and twenty-eight individuals were included in this alternative analysis, with 183 testing negative and 45 testing positive for TBI. Binary logistic regression was used to predict TBI test outcome using age, sex and TB incidence category of the participants’ country of birth as predictors. Odds ratios were examined to determine the impact of each variable. The binary logistic regression model was statistically significant, χ^2^ (4)=13.98, *p*=0.007, with a Nagelkerke R^2^ value of 0.09. The odds of a positive test were approximately 3.6 times higher for individuals born in a country classified as highly to severely endemic as compared to those born in countries classified as sub-endemic (OR=3.64; 95% CI: 1.26–10.55). Age was found to increase the odds of a positive TBI screen (OR=1.05; 95% CI: 1.02–1.09); that is, every additional year of age increased the odds of a positive screen by 1.05 times. In other words, the odds of obtaining a positive screen increased by 5% for every additional year a participant was aged. Neither participant sex, nor being born in a TB endemic country, was found to be statistically significant (Table A1).

**Table A1:** Logistic regression analysis data of participants arriving between 2019–2023

Predictor variable	ß	SE	*p*-value	Odds	95% CI for odds ratio	
Lower	Upper
Age	0.051	0.018	**0.004**	1.053	1.017	1.090
Sex	−0.258	0.347	0.458	0.773	0.391	1.526
Endemic	0.592	0.386	0.126	1.807	0.847	3.854
Highly to severely endemic	1.292	0.543	**0.017**	3.641	1.256	10.550

Abbreviations: CI, confidence interval; SE, standard error

Note: Model χ^2^=13.98, *p*=0.007, Nagelkerke R^2^=0.09, N=264. The dependent variable in the analysis was coded as 0=negative result and 1=positive result. Bolded *p*-values are statistically significant
